# Dehydro­leucodin: a guaiane-type sesquiterpene lactone

**DOI:** 10.1107/S1600536811048938

**Published:** 2011-11-30

**Authors:** Horacio A. Priestap, Khalil A. Abboud, Alvaro E. Velandia, Luis A. Lopez, Manuel A. Barbieri

**Affiliations:** a Department of Biological Sciences, Florida International University, Miami, FL 33199, USA; b Department of Chemistry, University of Florida, PO Box 117200 Gainesville, Gainesville, FL 32611-7200, USA; c Laboratory of Cytoskeleton and Cell Cycle, Instituto de Histología y Embriología, Facultad de Ciencias Médicas, Universidad Nacional de Cuyo, 5500 Mendoza, Argentina

## Abstract

Dehydro­leucodin [systematic name: (1*S*,6*S*,2*R*)-9,13-dimeth­yl-5-methyl­ene-3-oxatricyclo­[8.3.0.0^2,6^]trideca-9,12-diene-4,11-dione], C_15_H_16_O_3_, is a guanolide isolated from *Artemisia douglasiana*. The fused-ring system contains a seven-membered ring that adopts a chair conformation, a fused planar cyclo­pentenone ring and a five-membered lactone ring fused in envelope conformation. The absolute structure determined by X-ray analysis agrees with that previously assigned to this compound by NMR studies [Bohlmann & Zdero (1972 ▶). *Tetra­hedron Lett.* **13**, 621–624] and also with that of leucodine, a closely related guaianolide [Martinez *et al.* (1988 ▶). *J. Nat. Prod.* **51**, 221–228].

## Related literature

For NMR studies of dehydro­leucodin and leucodine, see: Bohlmann & Zdero (1972 ▶); Martinez *et al.*, (1988 ▶). For the pharmacological activity of dehydro­leucodin and related compounds, see Giordano *et al.* (1992 ▶).
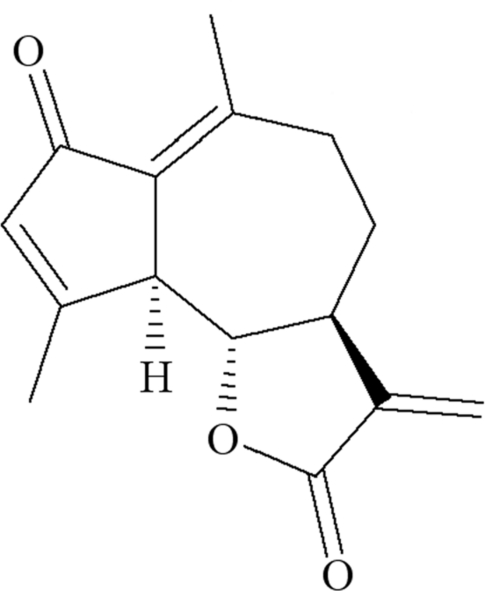


## Experimental

### 

#### Crystal data

C_15_H_16_O_3_
*M* *_r_* = 244.28
Orthorhombic, 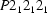

*a* = 7.5101 (3) Å
*b* = 11.1065 (4) Å
*c* = 15.0228 (6) Å
*V* = 1253.07 (8) Å^3^
*Z* = 4
Cu *K*α radiation
μ = 0.73 mm^−1^
*T* = 100 K
0.29 × 0.07 × 0.05 mm

#### Data collection

Bruker APEXII DUO diffractometer
Absorption correction: integration (*SADABS*; Bruker, 2008 ▶) *T*_min_ = 0.820, *T*_max_ = 0.962
10896 measured reflections
2166 independent reflections
2150 reflections with *I* > 2σ(*I*)
*R*_int_ = 0.064

#### Refinement

*R*[*F* ^2^ > 2σ(*F* ^2^)] = 0.027
*wR*(*F* ^2^) = 0.068
*S* = 1.05
2166 reflections
165 parameters
H-atom parameters constrained
Δρ_max_ = 0.19 e Å^−3^
Δρ_min_ = −0.14 e Å^−3^
Absolute structure: Flack (1983 ▶), 879 Friedel pairs
Flack parameter: 0.00 (17)

### 

Data collection: *APEX2* (Bruker, 2008 ▶); cell refinement: *SAINT* (Bruker, 2008 ▶); data reduction: *SAINT*; program(s) used to solve structure: *SHELXTL* (Sheldrick, 2008 ▶); program(s) used to refine structure: *SHELXTL*; molecular graphics: *SHELXTL*; software used to prepare material for publication: *SHELXTL*.

## Supplementary Material

Crystal structure: contains datablock(s) I, global. DOI: 10.1107/S1600536811048938/bg2432sup1.cif

Structure factors: contains datablock(s) I. DOI: 10.1107/S1600536811048938/bg2432Isup2.hkl

Supplementary material file. DOI: 10.1107/S1600536811048938/bg2432Isup3.mol

Supplementary material file. DOI: 10.1107/S1600536811048938/bg2432Isup4.cml

Additional supplementary materials:  crystallographic information; 3D view; checkCIF report

## Figures and Tables

**Fig. 1.**
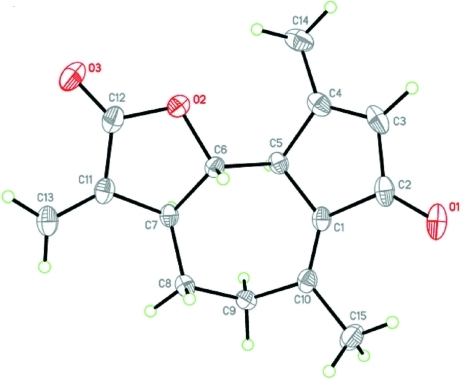
The molecular structure of the title molecule, showing 50% probability displacement ellipsoids.

## supplementary crystallographic information

### Comment

The title compound, a guaiane-type sesquiterpene lactone, was isolated from *Artemisia douglasiana* Bess (Asteraceae). NMR studies have been reported previously (Bohlmann & Zdero, 1972). By using a lanthanide shift reagent [Eu(fod)3] the lower field signals of dehydroleucodin could be resolved and showed the 5S, 6*R* and 7S configurations at the chiral centers. Here we report the crystal structure of dehydroleucodin that resulted coherent with the absolute stereochemistry previously reported by Bohlmann and Zdero (1972). The molecular geometry of dehydroleucodin is illustrated in Fig. 1. Inspection of the crystal structure shows that the cyclopentenone carbons, C-9 and C-10 are almost coplanar. The seven-membered ring adopts approximately a chair conformation with the atoms C-6, C-7, and C-8 above the plane. The lactone ring shows a half-chair conformation. H-5 and H-7 are located below the plane (beta-orientation) whereas H-6 is above the plane (beta-orientation), hence the configurations at the chiral centers 5, 6 and 7, is confirmed as being S, *R* and S, respectively. Bond distances and bond angles are normal.

### Experimental

Aerial parts of *Artemisia douglasiana* were collected in San Carlos, Mendoza (Argentina). The dried crushed plant material (10 g, dry weight) was exhaustedly extracted with boiling CHCl_3_. The CHCl_3_ extract was chromatographed on silica gel and alumina columns using mixtures of ethyl acetate and chloroform as eluants to give white crystals of dehydroleucodin (70 mg). This compound was identified by comparing the spectroscopic data with the previously published data (Bohlmann and Zdero, 1972). Crystals suitable for X-ray analysis were obtained by recrystallization from DMSO-water at 277K.

### Refinement

All the H atoms were placed in idealized positions and refined riding on their parent atoms, with C—H = 0.93-0.99 Å and *U*_iso_(H) =1.5*U*_eq_(C) for the methyl H atoms and 1.2*U*_eq_(C) for the remaining ones. The Flack *x* parameter is 0.00 (17) confirming that the correct enantiomer is being reported.

### Crystal data

**Table:**

C_15_H_16_O_3_	Dehydroleucodin
*M**_r_* = 244.28	*D*_x_ = 1.295 Mg m^−^^3^
Orthorhombic, *P*2_1_2_1_2_1_	Cu *K*α radiation, λ = 1.54178 Å
Hall symbol: P 2ac 2ab	Cell parameters from 9973 reflections
*a* = 7.5101 (3) Å	θ = 2.9–67.8°
*b* = 11.1065 (4) Å	µ = 0.73 mm^−^^1^
*c* = 15.0228 (6) Å	*T* = 100 K
*V* = 1253.07 (8) Å^3^	Needles, colourless
*Z* = 4	0.29 × 0.07 × 0.05 mm
*F*(000) = 520	

### Data collection

**Table:**

Bruker APEXII DUO diffractometer	2166 independent reflections
Radiation source: IµS microsource	2150 reflections with *I* > 2σ(*I*)
graphite	*R*_int_ = 0.064
phi and ω scans	θ_max_ = 66.4°, θ_min_ = 5.0°
Absorption correction: integration (*SADABS*; Bruker, 2008)	*h* = −8→7
*T*_min_ = 0.820, *T*_max_ = 0.962	*k* = −13→12
10896 measured reflections	*l* = −17→17

### Refinement

**Table:**

Refinement on *F*^2^	Secondary atom site location: difference Fourier map
Least-squares matrix: full	Hydrogen site location: inferred from neighbouring sites
*R*[*F*^2^ > 2σ(*F*^2^)] = 0.027	H-atom parameters constrained
*wR*(*F*^2^) = 0.068	*w* = 1/[σ^2^(*F*_o_^2^) + (0.0273*P*)^2^ + 0.2735*P*] where *P* = (*F*_o_^2^ + 2*F*_c_^2^)/3
*S* = 1.05	(Δ/σ)_max_ < 0.001
2166 reflections	Δρ_max_ = 0.19 e Å^−^^3^
165 parameters	Δρ_min_ = −0.14 e Å^−^^3^
0 restraints	Absolute structure: Flack (1983), 879 Friedel pairs
Primary atom site location: structure-invariant direct methods	Flack parameter: 0.00 (17)

### Special details

**Table:**

Geometry. All e.s.d.'s (except the e.s.d. in the dihedral angle between two l.s. planes) are estimated using the full covariance matrix. The cell e.s.d.'s are taken into account individually in the estimation of e.s.d.'s in distances, angles and torsion angles; correlations between e.s.d.'s in cell parameters are only used when they are defined by crystal symmetry. An approximate (isotropic) treatment of cell e.s.d.'s is used for estimating e.s.d.'s involving l.s. planes.
Refinement. All H atoms were positioned geometrically (C—H=0.93/1.00 Å) and allowed to ride with *U*_iso_(H)=1.2/1.5*U*_eq_(C). Methyl ones were allowed to rotate around the corresponding C—C. The Flack *x* parameter is 0.00 (17) confirming that the correct enantiomer is refined for this structure.

### Fractional atomic coordinates and isotropic or equivalent isotropic displacement parameters (Å^2^)

**Table:**

	*x*	*y*	*z*	*U*_iso_*/*U*_eq_	
O1	0.81504 (13)	−0.02962 (8)	0.26751 (7)	0.0312 (2)	
O2	0.80476 (13)	0.43297 (8)	0.09948 (6)	0.0248 (2)	
O3	0.87294 (16)	0.62331 (10)	0.06281 (7)	0.0411 (3)	
C1	0.68912 (15)	0.17287 (10)	0.25112 (8)	0.0192 (3)	
C2	0.77553 (16)	0.05869 (11)	0.22172 (10)	0.0240 (3)	
C3	0.79958 (18)	0.06797 (12)	0.12543 (10)	0.0278 (3)	
H3A	0.8526	0.0072	0.0897	0.033*	
C4	0.73794 (16)	0.17220 (12)	0.09389 (9)	0.0241 (3)	
C5	0.66907 (16)	0.25183 (10)	0.16885 (8)	0.0192 (3)	
H5A	0.5406	0.2712	0.1588	0.023*	
C6	0.77492 (16)	0.36796 (10)	0.18300 (8)	0.0183 (3)	
H6A	0.8926	0.3478	0.2104	0.022*	
C7	0.68007 (16)	0.46068 (10)	0.24137 (8)	0.0190 (3)	
H7A	0.5548	0.4662	0.2191	0.023*	
C8	0.66895 (18)	0.43149 (11)	0.33978 (8)	0.0227 (3)	
H8A	0.6197	0.5015	0.3723	0.027*	
H8B	0.7899	0.4154	0.3631	0.027*	
C9	0.55009 (17)	0.32097 (11)	0.35585 (8)	0.0224 (3)	
H9A	0.5127	0.3202	0.4190	0.027*	
H9B	0.4414	0.3286	0.3190	0.027*	
C10	0.63981 (17)	0.20247 (11)	0.33441 (9)	0.0209 (3)	
C11	0.77282 (17)	0.57383 (11)	0.21226 (9)	0.0215 (3)	
C12	0.82427 (19)	0.55285 (12)	0.11838 (9)	0.0269 (3)	
C13	0.80965 (17)	0.67457 (11)	0.25533 (10)	0.0266 (3)	
H13A	0.8719	0.7376	0.2260	0.032*	
H13B	0.7739	0.6839	0.3156	0.032*	
C14	0.7232 (2)	0.20714 (14)	−0.00173 (9)	0.0321 (3)	
H14A	0.7834	0.1469	−0.0387	0.048*	
H14B	0.7792	0.2859	−0.0107	0.048*	
H14C	0.5973	0.2116	−0.0186	0.048*	
C15	0.6668 (2)	0.12277 (12)	0.41396 (9)	0.0304 (3)	
H15A	0.5508	0.0977	0.4374	0.046*	
H15B	0.7319	0.1671	0.4600	0.046*	
H15C	0.7353	0.0515	0.3964	0.046*	

### Atomic displacement parameters (Å^2^)

**Table:**

	*U*^11^	*U*^22^	*U*^33^	*U*^12^	*U*^13^	*U*^23^
O1	0.0261 (5)	0.0160 (4)	0.0516 (6)	0.0001 (4)	−0.0029 (4)	0.0034 (4)
O2	0.0299 (5)	0.0231 (4)	0.0214 (4)	−0.0035 (4)	0.0023 (4)	0.0021 (4)
O3	0.0564 (7)	0.0322 (6)	0.0347 (6)	−0.0113 (5)	0.0045 (5)	0.0112 (5)
C1	0.0158 (6)	0.0158 (5)	0.0261 (6)	−0.0017 (5)	−0.0029 (5)	0.0001 (5)
C2	0.0158 (6)	0.0161 (6)	0.0401 (8)	−0.0039 (5)	−0.0027 (5)	−0.0029 (5)
C3	0.0244 (7)	0.0213 (6)	0.0377 (8)	−0.0010 (6)	0.0033 (6)	−0.0118 (6)
C4	0.0191 (6)	0.0257 (6)	0.0275 (7)	−0.0051 (5)	0.0011 (5)	−0.0076 (6)
C5	0.0169 (6)	0.0182 (6)	0.0225 (6)	−0.0011 (5)	−0.0013 (5)	−0.0027 (5)
C6	0.0186 (6)	0.0176 (6)	0.0186 (6)	−0.0002 (5)	−0.0010 (5)	0.0015 (5)
C7	0.0179 (6)	0.0156 (5)	0.0236 (6)	0.0019 (5)	−0.0008 (5)	−0.0001 (5)
C8	0.0275 (7)	0.0179 (6)	0.0227 (7)	0.0003 (5)	−0.0003 (5)	−0.0032 (5)
C9	0.0259 (6)	0.0215 (6)	0.0198 (6)	−0.0015 (6)	0.0017 (5)	−0.0022 (5)
C10	0.0193 (6)	0.0181 (6)	0.0253 (6)	−0.0041 (5)	−0.0037 (5)	0.0014 (5)
C11	0.0176 (6)	0.0179 (6)	0.0289 (7)	0.0018 (5)	−0.0032 (5)	0.0037 (5)
C12	0.0277 (7)	0.0223 (6)	0.0305 (7)	−0.0047 (6)	−0.0033 (6)	0.0050 (5)
C13	0.0219 (6)	0.0190 (6)	0.0389 (7)	0.0007 (5)	−0.0039 (6)	0.0003 (6)
C14	0.0315 (8)	0.0399 (8)	0.0251 (7)	−0.0044 (6)	0.0018 (6)	−0.0099 (6)
C15	0.0371 (8)	0.0252 (6)	0.0288 (7)	−0.0026 (6)	−0.0048 (6)	0.0075 (6)

### Geometric parameters (Å, °)

**Table:**

O1—C2	1.2343 (17)	C7—H7A	1.0000
O2—C12	1.3692 (16)	C8—C9	1.5369 (17)
O2—C6	1.4649 (14)	C8—H8A	0.9900
O3—C12	1.2012 (17)	C8—H8B	0.9900
C1—C10	1.3456 (19)	C9—C10	1.5132 (17)
C1—C2	1.4914 (16)	C9—H9A	0.9900
C1—C5	1.5230 (17)	C9—H9B	0.9900
C2—C3	1.461 (2)	C10—C15	1.5009 (18)
C3—C4	1.334 (2)	C11—C13	1.3218 (18)
C3—H3A	0.9500	C11—C12	1.4808 (19)
C4—C14	1.4920 (19)	C13—H13A	0.9500
C4—C5	1.5225 (17)	C13—H13B	0.9500
C5—C6	1.5299 (16)	C14—H14A	0.9800
C5—H5A	1.0000	C14—H14B	0.9800
C6—C7	1.5286 (17)	C14—H14C	0.9800
C6—H6A	1.0000	C15—H15A	0.9800
C7—C11	1.5019 (16)	C15—H15B	0.9800
C7—C8	1.5158 (17)	C15—H15C	0.9800
			
C12—O2—C6	108.55 (9)	C7—C8—H8B	109.5
C10—C1—C2	127.08 (12)	C9—C8—H8B	109.5
C10—C1—C5	125.92 (11)	H8A—C8—H8B	108.1
C2—C1—C5	107.0 (1)	C10—C9—C8	113.74 (10)
O1—C2—C3	125.31 (13)	C10—C9—H9A	108.8
O1—C2—C1	127.97 (13)	C8—C9—H9A	108.8
C3—C2—C1	106.68 (11)	C10—C9—H9B	108.8
C4—C3—C2	111.72 (12)	C8—C9—H9B	108.8
C4—C3—H3A	124.1	H9A—C9—H9B	107.7
C2—C3—H3A	124.1	C1—C10—C15	123.99 (12)
C3—C4—C14	126.41 (12)	C1—C10—C9	122.21 (11)
C3—C4—C5	111.05 (12)	C15—C10—C9	113.80 (11)
C14—C4—C5	122.40 (12)	C13—C11—C12	123.01 (12)
C4—C5—C1	103.43 (10)	C13—C11—C7	131.52 (13)
C4—C5—C6	114.58 (10)	C12—C11—C7	105.47 (10)
C1—C5—C6	108.75 (9)	O3—C12—O2	121.46 (13)
C4—C5—H5A	110.0	O3—C12—C11	129.70 (13)
C1—C5—H5A	110.0	O2—C12—C11	108.82 (11)
C6—C5—H5A	110.0	C11—C13—H13A	120.0
O2—C6—C7	103.33 (9)	C11—C13—H13B	120.0
O2—C6—C5	112.09 (9)	H13A—C13—H13B	120.0
C7—C6—C5	113.92 (10)	C4—C14—H14A	109.5
O2—C6—H6A	109.1	C4—C14—H14B	109.5
C7—C6—H6A	109.1	H14A—C14—H14B	109.5
C5—C6—H6A	109.1	C4—C14—H14C	109.5
C11—C7—C8	119.24 (11)	H14A—C14—H14C	109.5
C11—C7—C6	100.4 (1)	H14B—C14—H14C	109.5
C8—C7—C6	116.17 (10)	C10—C15—H15A	109.5
C11—C7—H7A	106.7	C10—C15—H15B	109.5
C8—C7—H7A	106.7	H15A—C15—H15B	109.5
C6—C7—H7A	106.7	C10—C15—H15C	109.5
C7—C8—C9	110.86 (10)	H15A—C15—H15C	109.5
C7—C8—H8A	109.5	H15B—C15—H15C	109.5
C9—C8—H8A	109.5		
